# Preliminary clinical testing to inform development of the Critical Care Pain Observation Tool for Families (CPOT-Fam)

**DOI:** 10.1080/24740527.2023.2235399

**Published:** 2023-09-14

**Authors:** Anmol Shahid, Bonnie G. Sept, Victoria S. Owen, Corson Johnstone, Rameiya Paramalingam, Stephana J. Moss, Rebecca Brundin-Mather, Karla D. Krewulak, Andrea Soo, Jeanna Parsons-Leigh, Céline Gélinas, Kirsten M. Fiest, Henry T. Stelfox

**Affiliations:** a Department of Critical Care Medicine, Cumming School of Medicine, University of Calgary & Alberta Health Services, Calgary, Alberta, Canada; b School of Health Administration, Faculty of Health, Dalhousie University, Halifax, Nova Scotia, Canada; c Ingram School of Nursing, McGill University, and Centre for Nursing Research and Lady Davis Institute, Jewish General Hospital–CIUSSS West-Central Montreal, Montreal, Quebec, Canada; d Department of Psychiatry and Hotchkiss Brain Institute, Cumming School of Medicine, University of Calgary, Calgary, Alberta, Canada; e Department of Community Health Sciences and O’Brien Institute for Public Health, Cumming School of Medicine, University of Calgary, Calgary, Alberta, Canada

**Keywords:** Pain assessment, critical care pain observation tool, family engagement, intensive care unit pain, tool development, quality improvement

## Abstract

**Introduction:**

Many patients in the intensive care unit (ICU) cannot communicate. For these patients, family caregivers (family members/close friends) could assist in pain assessment. We previously adapted the Critical Care Pain Observation Tool (CPOT) for family caregiver use (CPOT-Fam). In this study, we conducted preliminary clinical evaluation of the CPOT-Fam to inform further tool development.

**Methods:**

For preliminary testing, we collected (1) pain assessments of patients in the ICU from family caregivers (CPOT-Fam) and nurses (CPOT) and determined the degree of agreement (kappa coefficient, κ) and (2) collected openended feedback on the CPOT-Fam from family caregivers. For refinement, we used preliminary testing data to refine the CPOT-Fam with a multidisciplinary working group.

**Results:**

We assessed agreement between family caregiver and nurse pain scores for 29 patients. Binary agreement (κ) between CPOT-Fam and CPOT item scores (scores ≥2 considered indicative of significant pain) was fair, κ = 0.43 (95% confidence interval [CI] 0.18–0.69). Agreement was highest for the CPOT-Fam items ventilator compliance/vocalization (weighted κ = 0.48, 95% CI 0.15–0.80) and lowest for muscle tension (weighted κ = 0.10, 95% [CI] −0.17 to 0.20). Most participants (*n* = 19; 69.0%) reported a very positive experience using the CPOT-Fam, describing it as “good” and “easy-to-use/clear/straightforward.” We iteratively refined the CPOT-Fam over five cycles using the data collected until no further revisions were suggested.

**Conclusion:**

Our preliminary clinical testing suggests that family involvement in pain assessment in the ICU is well perceived. The CPOT-Fam has been further refined and is now ready for clinical pilot testing to determine its feasibility and acceptability.

## RÉSUMÉ

**Introduction:** De nombreux patients de l’unité de soins intensifs (USI) ne peuvent pas communiquer. Pour cespatients, les aidants familiaux (membres de la famille/amis proches) pourraient aider à l’évaluation de la douleur. Nous avons précédemment adapté l’outil d’observation de la douleur en soins intensifs (CPOT) pour qu’il puisse être utilisé par des aidants familiaux (CPOTFam). Dans cette étude, nous avons mené une évaluation clinique préliminaire du CPOT-Fam afin d’éclairer davantage le développement de l’outil.

**Méthodes:** Pour les tests préliminaires, nous avons recueilli (1) des évaluations de la douleur des patients en unité de soins intensifs auprès d’aidants familiaux (CPOT-Fam) et d’infirmières (CPOT) et déterminé le degré de concordance (coefficient de Kappa, κ) et (2) des commentaires ouverts sur le CPOT-Fam auprès d’aidants familiaux. Nous avons ensuite utilisé les données des tests préliminaires pour affiner le CPOT-Fam avec un groupe de travail multidisciplinaire.

**Résultats:** Nous avons évalué la concordance entre les scores obtenus par des aidants familiaux et des infirmières pour les énoncés portant sur la douleur pour 29 patients. La concordance binaire (κ) entre les scores obtenus pour les énoncés du CPOT-Fam et du CPOT (un score ≥ 2 était considéré comme un indicateur de douleur importante) était passable, κ = 0,43 (intervalle de confiance à 95 % [IC] 0,18-0,69). La concordance était la plus élevée pour les énoncés du CPOT-Fam portant sur l’observance de la ventilation/vocalisation (κ pondéré = 0,48, IC à 95 % 0,15-0,80) et la plus faible pour la tension musculaire (κ pondéré = 0,10, 95% [IC] − 0,17 à 0,20). La plupart des participants (n = 19; 69,0 %) ont fait état d’une expérience très positive de l’utilisation du CPOT-Fam, le décrivant comme « bon » et « facile à utiliser/clair/simple. » Nous avons affiné le CPOT-Fam de manière itérative sur cinq cycles en utilisant les données recueillies jusqu’à ce qu’aucune autre révision ne soit suggérée.

**Conclusion:** Nos tests cliniques préliminaires indiquent que la participation de la famille à l’évaluation de la douleur dans l’unité de soins intensifs est bien perçue. Le CPOT-Fam a été affiné et est maintenant prêt pour le test clinique pilote afin de déterminer sa faisabilité et son acceptabilité.

## Introduction

Family caregivers (e.g., significant others, parents, children, friends) can identify patient behaviors and expressions indicating the presence or severity of pain based on their intimate knowledge of the patient.^1,2^ This may be helpful in the intensive care unit (ICU) where many patients are unable to self-report pain due to their clinical condition.^3–6^ It has been found that patients’ self-reported pain ratings are in closer agreement with family caregivers’ pain ratings than with nurses and physicians.^1,7^ This suggests that involving family caregivers in assessing the pain of patients in the ICU who are unable to self-report could improve pain recognition.

The Critical Care Pain Observation Tool (CPOT) is widely used by bedside nurses to assess pain in patients in the ICU who are unable to self-report their pain.^8–10^ The CPOT uses four pain behavior items (facial expression, body movements, compliance with ventilator [if the patient is intubated] or vocalization [if the patient is not intubated], and muscle tension) to assess pain.^11,12^ Each item is scored from 0 to 2 (0 being the lowest score) representing the intensity of pain-related behaviors. The scores are additive for a maximum value of 8 and a threshold score ≥2 is interpreted as significant pain, triggering a clinical intervention for pain management.^13^ The CPOT has shown moderate correlations (intraclass correlation coefficients [ICCs] of 0.59 and 0.71 from two different studies) to self-reported pain intensity during painful procedures in the ICU (such as turning) and moderate sensitivity (86%) and specificity (78%) when used by ICU nurses to assess pain in patients in the ICU.^10,11^

Although the CPOT was designed for use by nurses, it has shown potential for use by family caregivers provided training opportunities.^12^ A recent study observing family caregivers using the CPOT to assess pain in patients in the ICU reported the tool’s potential to empower families by (1) confirming their observations of pain behaviors, (2) allowing more focus on the patient, and (3) advocating for better pain management.^12^ However, this study also suggested that family caregivers’ understanding of pain may not align with some behavioral items of the CPOT (e.g., many family caregivers considered the compliance with ventilator item less relevant to their understanding of pain).^12^ Family caregivers’ understandings of these behavioral items may have been limited owing to the CPOT’s terminology and their unfamiliarity with ICU technology and alarms, which are intended for interpretation by clinicians with relevant knowledge and skills.^11^

Considering that family caregivers may be able to facilitate pain assessment for patients unable to self-report pain, it is important to provide them with appropriate tools to support them in this role.^6^ For this purpose, we previously adapted the CPOT to create the CPOT-Fam by (1) simplifying the terminology used in the CPOT, (2) creating illustrations to represent CPOT scoring options, (3) restructuring the CPOT for easier use by family caregivers, and (4) conducting preclinical testing with public participants.^14^

To further develop the CPOT-Fam, we aimed to:

1a. Obtain pain assessments from family caregivers (CPOT-Fam) and nurses (CPOT) of patients in the ICU.

1b. Collect open-ended feedback on the CPOT-Fam from families who had used the tool.

2. Have a multidisciplinary working group refine the CPOT-Fam using the data collected.

## Methods

Ethical approval to conduct this study was obtained from the University of Calgary Conjoint Health Research Ethics Board (REB 21–0748) alongside a research agreement with the health custodian at Alberta Health Services. We obtained permission to adapt the CPOT for family use from the American Association of Critical Care Nurses and have detailed specific tool adaption methodology in previously published manuscripts.^14,15^ We adapted the CPOT for family use by replacing clinical jargon with simple terms, adding illustrations, and ensuring that the adapted tool was suitable for layperson use through the Patient Education Materials Assessment Tool.

### Phases 1a and 1b: CPOT-Fam Preliminary Clinical Testing

**Figure A1.**
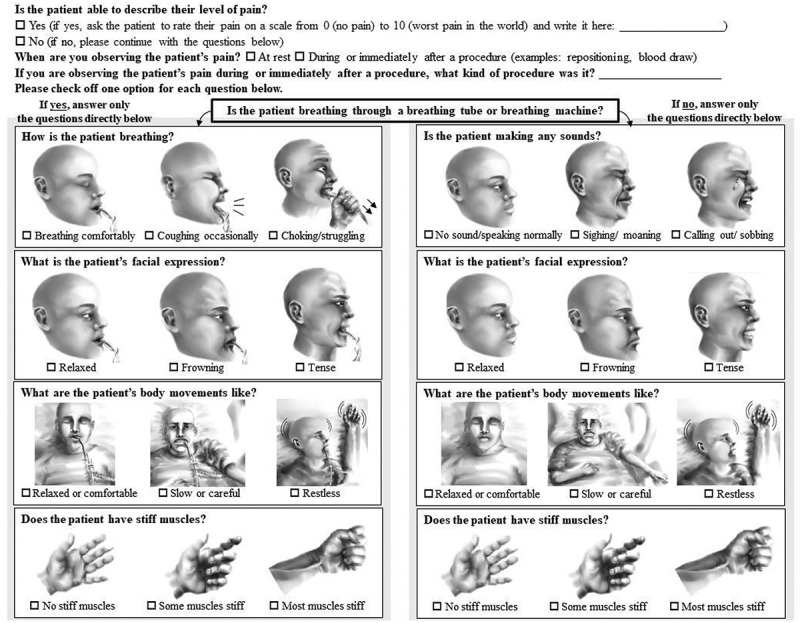
A version of the CPOT-Fam used in the study.

Family caregivers of patients in the ICU were invited to use the CPOT-Fam (Figure A1) to assess pain in patients in the ICU and provide feedback on the tool. Agreement between family caregiver pain scores (CPOT-Fam) and nurse pain scores (CPOT) was evaluated.

#### Study Setting, Participants, and Recruitment

We invited family caregivers of patients admitted to the Foothills Medical Center medical–surgical ICU in Calgary, Alberta, Canada, to participate in this study (March 22 to May 10, 2022). Family caregivers were eligible for inclusion if they were over 18 years of age, could communicate in English (i.e., understand, read, speak), and could provide informed consent. Consent was obtained in writing using recruitment documentation that ensured that potential participants were provided adequate information about the study and understood their role.

#### Procedures

Study participants were asked to complete the paper-based CPOT-Fam for their critically ill family member while at rest (when no clinical procedures or mobilizations/repositionings were being done) and provide feedback on the tool. Study participants were also asked to complete a paper-based survey of demographic information including their age, sex, level of education, and relationship to the critically ill patient. Study procedures and instruments used (including the demographic survey) are described in further detail in a previously published protocol manuscript.^15^ The research team (A.S., B.G.S., C.J.) facilitated participants’ completion of the CPOT-Fam and surveys by being available in person to answer questions. All data were stored securely on a University of Calgary–approved digital location in accordance with institutional procedures.

After collection of information from family participants, the closest time-stamped CPOT score by an ICU nurse (documented every 4 h as part of routine bedside care) was abstracted from eCritical, a population-based provincial critical care clinical information system that captures demographic, clinical, and outcomes data for all admitted patients in the ICU, including notes on sedation and pain.^19^ CPOT scores were collected from eCritical to minimize additional workload for nurses, given the burden of the COVID-19 pandemic at the time of data collection. Notes on sedation and pain were reviewed to establish context for each CPOT score. Any pain scores assigned within 30 min following a clinical procedure (determined by reviewing the notes) were excluded because clinical procedures can impact pain scores.^13^

#### Data Analysis

Quantitative data collected from participants was analyzed using Microsoft Excel v16.0. Participant characteristics and responses to closed-ended questions (e.g., multiple choice, yes/no) were compiled into counts and percentages. Kappa, weighted kappa, and percentage agreement were used to determine agreement between family caregiver pain scores (CPOT-Fam) and the closest time-stamped nurse scores (CPOT) for each patient.^16^ Agreement was interpreted using standard recommendations (0.81–1.0: excellent agreement, 0.61–0.80: good agreement, 0.41–0.60: moderate agreement, 0.21–0.40: slight agreement, and <0.21: poor agreement.^20^

Textual data collected on the follow-up survey (i.e., feedback on the CPOT-Fam on aspects like clarity, ease of use, and feedback on images and wording) were analyzed using NVivo 12. Inductive thematic analysis was completed by research team members (A.S., B.G.S., C.J.) who reviewed the complete data set and identified initial codes that represented categories of feedback (such as “clarity of CPOT-Fam wording,” “clarity of CPOT-Fam illustrations” “usefulness of CPOT-Fam”) received from study participants. The research team members then assigned every comment as either having “positive sentiment,” “neutral sentiment,” or “negative sentiment.” The research team (A.S., B.G.S., C.J.) members then compared sentiments and discussed converging and diverging opinions.^17^

#### Sample Size and Power Considerations

No formal sample size calculations were done because the purpose of this study was to preliminarily test the CPOT-Fam in clinical settings and use the agreement between family and nurse pain scores and feedback from family participants to refine the CPOT-Fam. A sample convenient for recruitment timelines and team availability was used.^18^

### Phase 2: CPOT-Fam Revision

A multidisciplinary working group was assembled by invitation to revise the CPOT-Fam. The working group comprised a patient partner with lived experience caring for critically ill family members (B.G.S.), critical care clinicians (nurse practitioners: V.S.O., C.G.; physician: H.T.S.), and critical care researchers with experience in pain assessment (C.G., A.S.), tool development (K.F., C.G.), and qualitative and/or quantitative methods and analyses (S.J.M., J.P.L., R.B.M., K.D.K., A.S.). C.G. developed the original CPOT and lent expertise on the process of tool development alongside a nursing perspective on family involvement in pain assessment.

The working group refined the CPOT-Fam based on the family–nurse agreement scores as well as family feedback on the CPOT-Fam. The working group identified a need for rapid cycle revisions. Additional family caregivers were recruited to provide further feedback on the CPOT-Fam (specifically any changes to illustrations and wording). Family caregiver feedback was discussed as it was received and all comments that pertained to improving clarity, ease of use, and ability to capture pain of the CPOT-Fam were discussed by the research team (T.S., A.S.) to determine how to best refine the CPOT-Fam accordingly. The CPOT-Fam was presented to the working group using a modified nominal group technique to ensure that the group was satisfied with the illustrations and wording used in each item of the CPOT-Fam and considered the tool suitable for future clinical pilot testing.^23^

## Results

### CPOT-Fam Usage in ICU Settings

**Figure 1.**
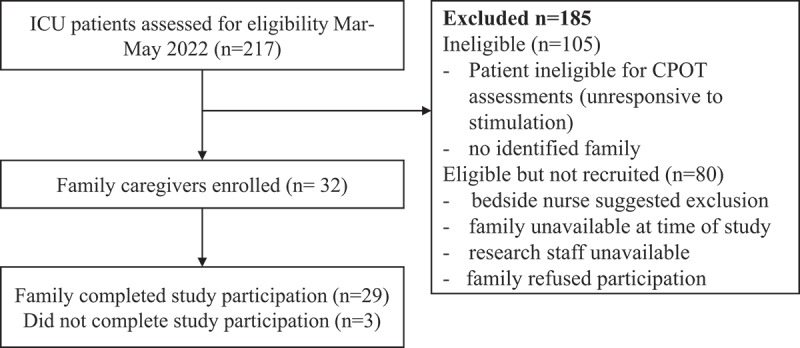
Flow diagram showing participant selection.

Of the 37 family caregivers approached to participate in the study, most consented (*n* = 32; 86.4%) and completed (*n* = 29; 78.3%) participation in the study (Figure 1). Most participants were female (*n* = 25; 86.2%), belonged to an English-speaking household (*n* = 25, 86.2%), and were college or university educated (*n* = 18; 62.1%). Eleven participants were significant others (38.0%) and 16 were first-degree relatives of the patient in the ICU (55.1%). Participant demographic characteristics are detailed in Table 1.

**Table 1.** Characteristics of family caregiver study participants (*N* = 29).

Characteristics	No. participants, *n* (%)
Age (years)	
20–39	5 (17.2)
40–59	10 (34.5)
60+	14 (48.3)
Female	25 (86.2)
Education	
High school diploma or less	5 (17.2)
Vocational/trade certification	6 (21.0)
Some college or university	6 (21.0)
College or university degree	9 (31.0)
Graduate or professional degree	3 (10.3)
Language spoken at home	
English	25 (86.2)
Other	4 (13.8)
Relationship with patient in the ICU	
Significant other	11 (38.0)
First degree relative (parent, child, sibling)	16 (55.1)
Other (distant relative, friend)	2 (7.0)

#### Agreement in CPOT-Fam Scoring

**Figure 2.**
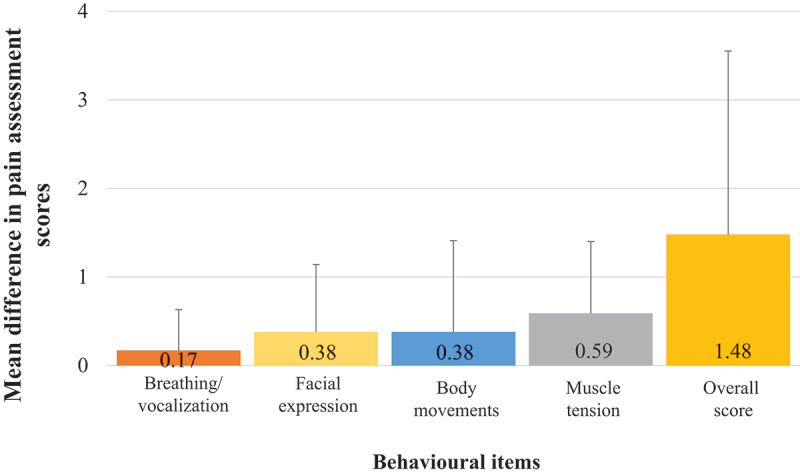
Mean difference in pain assessment scores (calculated as CPOT-Fam score – CPOT score). Bars represent standard deviation.

Twenty-nine paired CPOT-Fam and CPOT scores (one set of scores for each patient) were used to calculate agreement. Data characteristics of CPOT-Fam and CPOT, shown as the median (interquartile range) and minimum (min) and maximum (max) scores, were as follows: CPOT-Fam: 2.00 (0.00–4.50), min = 0, max = 6; CPOT: 0.00 (0.00–2.00), min = 0, max = 8. The median time difference (interquartile range) between CPOT-Fam scores by family caregivers and CPOT scores by nurses was 90.0 (87.0) minutes. Agreement (κ) between overall CPOT-Fam and CPOT scores for significant pain (defined as total score ≥2 versus no pain defined as total score<2) was a κ of 0.43 (95% confidence interval [CI] 0.18–0.69). Agreement was further calculated according to behavioral items of the tools (facial expression, body movements, ventilator compliance [if intubated] or vocalization [if not intubated], and muscle tension). Agreement was highest for the ventilator compliance/vocalization item. There was fair agreement for assessing body movements and poor agreement for facial expression and muscle tension items. The breakdown of agreement by each behavioral item is shown in Table 2. Average CPOT-Fam scores were higher than CPOT scores for every behavioral item. The distribution of differences in scores between the CPOT-Fam and CPOT across behavioral items is shown in Figure 2.

**Table 2.** Pain score agreement between families (CPOT-Fam) and nurses (CPOT).

Tool scoring items	Percent agreement^a^	Agreement (κ) [95% CI]^a^
Overall agreement (significant pain vs. pain)^b^	69.0	0.43 [0.18–0.69]
Ventilator compliance (if intubated) or vocalization (if not intubated)	75.9	0.48 [0.15–0.80]^c^
Facial expression	58.6	0.04 [−0.20–0.29]
Body movements	62.1	0.32 [0.05–0.60]
Muscle tension	41.4	0.10 [−0.17–0.20]

^a^Percent agreement and kappa (unweighted for overall agreement and weighted for ordinal subcategories) for behavioral items of pain assessment were generated through comparison of pain scores between families (CPOT-Fam) and nurses (CPOT) for a given patient.

^b^No pain was defined as scores below 2; pain warranting clinical intervention defined as scores of 2 or higher as per guidelines.^32^

^c^Represents moderate agreement, interpreted as per recommendations.^20^

#### Participant Feedback–Closed-Ended

All participants (*n* = 29; 100.0%) indicated that they would feel empowered to act if they identified pain in their family caregiver by either (1) contacting the care team or (2) using a nonmedical intervention like head rubbing to comfort the patient (Table 3). Most (19, 66.0%) participants indicated that they felt moderately or extremely comfortable in their ability to tell whether a loved one was experiencing pain, and 3 participants indicated that they felt moderately uncomfortable assessing the patient’s pain (10.3%; Table 3).

**Table 3.** Data summary of closed-ended items presented to family caregiver study participants.

Closed-ended items	Response options	Summary of responses
“If you identify pain in your family member, do you feel empowered to act on this information?”	“Yes” or “no”	*n* = 29 (100.0%) selected “yes”
“What would you do if you identified pain in your family member?”	“Contact the care team” or “other”	*n* = 23 (79.3%) selected “contact the care team” *n* = 6 (20.7%) selected “other,” further specifying “head rubbing” or “hand holding”
“How comfortable do you feel in your ability to tell whether your family member is experiencing pain?”	Five-point scale of *extremely comfortable* to *extremely uncomfortable*	*n* = 19 (66.0%) selected “moderately to extremely comfortable” *n* = 7 (24.1%) selected “not comfortable or uncomfortable” *n* = 3 (10.3%) selected “moderately uncomfortable”

#### Participant Feedback–Open-Ended

Twenty-seven participants provided a text response to the open-ended question: “Please describe your experience with using the pain detection method (i.e., CPOT-Fam) in a few words” (93.1%). Text responses were categorized according to feedback sentiment and presented as frequency (*n*) of positive experiences using the CPOT-Fam (*n* = 19), negative experiences using the CPOT-Fam (*n* = 2), and specific feedback for improvement (*n* = 6). Most of the positive feedback received was brief and used wording like “good” (*n* = 5; 26.3%) and “easy-to-use/clear/straightforward” (*n* = 9; 47.4%). Negative feedback (*n* = 2; 6.90%) received described the CPOT-Fam as “vague” and “[making it] difficult to classify pain.” Two participants stated that the CPOT-Fam could use more “variation” or “explanation” of pain behavior items, whereas others (*n* = 4; 13.8%) stated that the CPOT-Fam did not capture pain effectively in patients who are “sedated,” “sleeping” or exhibiting other potential signs of pain such as “involuntary twitching.” It should be noted that 2 family caregivers requested assistance from the research team (A.S., B.G.S., C.J.) to complete the CPOT-Fam (6.9%). These family caregivers wished to know which side of the CPOT-Fam to fill out and were able to complete the CPOT-Fam successfully after a brief explanation from the research team (A.S., B.G.S., C.J.).

### Revision of the CPOT-Fam

The working group met to discuss agreement between family and nurse pain scores and reflect on the feedback received from families. In a structured group meeting, the research lead (A.S.) presented agreement in pain scores and feedback received from the family and invited comments regarding how to improve the CPOT-Fam from each working group member sequentially. The comments were captured on an online note-taking platform and duplicate comments were removed. The working group then ranked the suggestions for revision in order of perceived priority.

During the first meeting, the working group agreed that the CPOT-Fam was mostly effective for family caregiver use and recommended changes to illustrations and wording to improve effectiveness and ease of use. Major illustration changes suggested by the working group were to (1) reorient the facial expression illustrations to face forward (originally faced sideways) and (2) exaggerate frowning and tense expressions in the facial expression category. The major wording changes recommended were to (1) remove all questions unrelated to the CPOT pain items (top of the page, shown in Figure A1), (2) modify some question prompts for some items of the CPOT-Fam to be statements rather than questions to mitigate any confusion about terminology (for example, the body movements prompt was changed to the following: “the patient appears to be … 1) relaxed/comfortable, 2) moving slowly/carefully, 3) restless/uncomfortable”), and (3) add details to capture individual patient behavior or states (e.g., adding in details like the “patient’s eyes may be open or closed”). The working group also recommended attaching numerical scores to the CPOT-Fam items (as in the CPOT) so that families could tally up their scores into a total numerical CPOT-Fam score.

The working group recommended conducting further clInical testing phases of the CPOT-Fam (original protocol proposed a single set of clinical evaluations).^15^ The working group strongly recommended that future evaluations coordinate the timing of family and nurse assessments for a more accurate representation of instantaneous pain scores (current evaluation was designed as a pragmatic study to leverage routine bedside assessments). Finally, the working group identified a need for rapid revisions of the CPOT-Fam and suggested recruiting additional family caregivers to provide detailed feedback on the tool.

**Figure 3.**
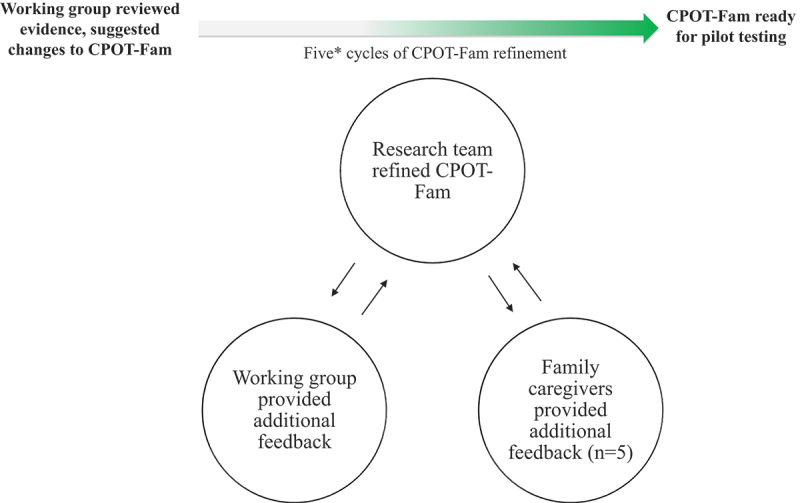
CPOT-Fam tool revision and refinement process. Working group included research team and external research and clinical collaborators. *Five total cycles of revision were completed (n = 3 between working group and research team; n = 2 between the research team, considering additional feedback from family caregivers noted above).

**Figure 4.**
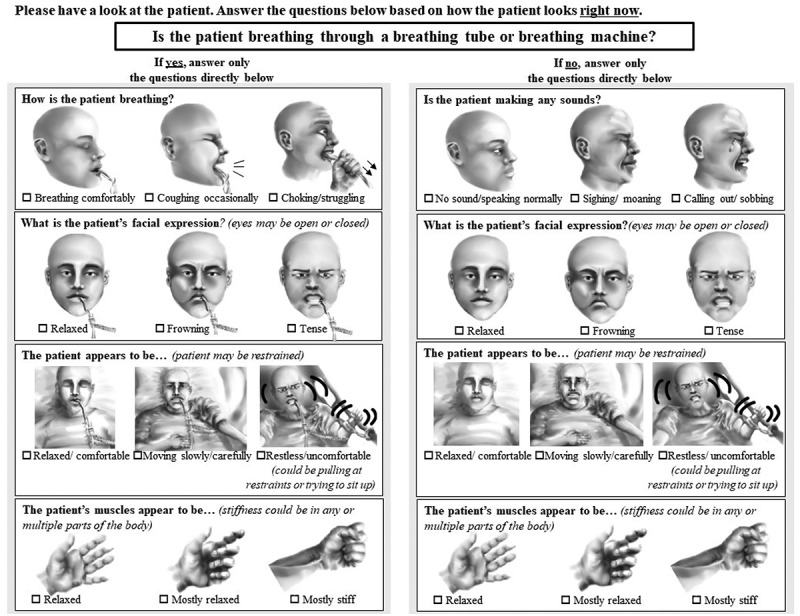
The CPOT-Fam, refined in preparation for clinical pilot testing.

After the initial working group meeting, the research lead (A.S.) refined the CPOT-Fam illustrations and wording. As suggested by the working group, feedback on the CPOT-Fam was collected from five additional family caregivers (agreement data from these participants are not included in the results presented). Additional family caregiver feedback was discussed as it was received and all comments that pertained to improving clarity (in words and images used), ease of use, and ability to capture pain of the CPOT-Fam were discussed by the research team (T.S., A.S.) to determine how to best refine the CPOT-Fam accordingly. Important feedback incorporated during the revision cycles included (1) addition of physical restraints in CPOT-Fam illustrations (evident in the body movements illustrations in Figure 4) and (2) removal of numerical scores in the CPOT-Fam (family caregivers indicated they found tallying numbers to be overwhelming). The CPOT-Fam refinement process is summarized in Figure 3. The refined CPOT-Fam was then circulated to the working group to solicit further feedback and a CPOT-Fam version ready for clinical pilot testing was established (Figure 4).

## Discussion

In this study, we conducted a preliminary clinical evaluation of a pain assessment tool adapted for use by family caregivers of critically ill patients (CPOT-Fam) in the ICU to identify areas for improvement. Considering the variability in agreement between individual items of the CPOT-Fam and CPOT, we found the overall agreement between family and nurse pain scores to be fair. Specifically, agreement was moderate for the ventilator compliance/vocalization items of the tool, slight for the body movements item of the tool, and poor for the facial expression and muscle tension items of the tool. Preliminary clinical testing also showed that the CPOT-Fam was viewed favorably by most study participants, who generally felt comfortable assessing pain in their critically ill loved one. The data collected were used by a multidisciplinary working group to iteratively refine the CPOT-Fam.

A small number of studies have examined pain assessments by family caregivers of critically ill patients. Among these, a large study of families assessing pain in critically ill patients showed that family caregivers can accurately estimate the presence of pain in patients in the ICU 73.5% of the time.^3,20^ Another study demonstrated that patient self-report pain scores have better agreement with family caregiver scores (ICC = 0.43) rather than nurse (ICC = 0.29) scores.^4^ The aforementioned studies noted that spouses of patients in the ICU tend to participate more in pain scoring than other family caregivers, suggesting that spouses may be more available for proxy pain assessment in the ICU.^21,22^ Studies in non-ICU settings strongly suggest that family caregivers are familiar with a patient’s individualistic pain behaviors and could be important in the assessment and management of pain.^22^ Studies in hospital settings outside of the ICU have reported differential effects of age and sex on pain score agreement between families and patients and higher agreement between spouse pain scores and patient pain scores in comparison to other family caregivers.^22^

Though family caregivers may be helpful in assessing pain in patients in the ICU who are unable to self-report, previous studies of clinician attitudes have shown that factors such as family dynamics, communication ability, and family health literacy can significantly hinder family engagement in the ICU.^23^ Interestingly, as shown in our study’s distribution of differences in pain scores between family caregivers and nurses (family caregiver pain ratings were higher for all CPOT behavioral items), previous ICU-based studies have also reported that family caregivers have a tendency to provide higher estimates of pain in their loved ones.^19,20^ This could be a result of varying health literacy or high anxiety experienced by family caregivers of patients in the ICU, which could lead to disagreements between family and clinicians and affect care plans.^23^ This challenge could be proactively addressed by acquiring bedside nurse viewpoints on involving family caregivers in pain assessment of patients. Furthermore, because bedside nurses spend a significant amount of time with the patient, they may have important insights into how the CPOT-Fam can be improved to better capture pain. Preliminary clinical testing of the CPOT-Fam has shown that there is room for improvement.

### Limitations

The largest limitation of the study was its pragmatic design. Nurse pain scores (CPOT) were obtained as part of routine patient care and documented in an electronic information system. In many instances, the time difference between nurse and family pain scores was long enough to allow for potentially important changes in the patient’s clinical state (e.g., the patient had a procedure done or received pain medication). This could have affected the agreement between family caregiver and nurse pain scores. An additional limitation arising from the pragmatic design of this study was the collection of CPOT-Fam assessments when pain behaviors may be minimal (i.e., at rest). Further work will be needed to evaluate the completion of the CPOT-Fam around potentially painful procedures (i.e., not at rest). Another limitation to this study was the absence of a reference standard of pain, because the patients included in the study were not able to self-report pain. Although we learned of family experiences with using the CPOT-Fam and calculated agreement between family and nurse pain assessments, we cannot comment on which pain assessment (family or nurse) is more closely aligned with the patient’s experience. Furthermore, some family caregivers’ characteristics (i.e., health care experiences as a patient, prior experiences as a caregiver, level of education) may have affected their use of the CPOT-Fam. Because we did not investigate family caregiver characteristics owing to a modest sample size, this can be considered a limitation to our study and will be addressed in subsequent evaluations of the CPOT-Fam. Finally, because the CPOT-Fam is not designed for use with patients deemed unresponsive to stimulation, our inclusion criteria omitted these patients, and we are unable to comment on pain assessment in this population.

## Conclusions

In this study, we preliminarily tested the CPOT-Fam in the ICU environment to determine the agreement between family and nurse pain scores and collected feedback from families. The CPOT-Fam was further refined based on the feedback from family caregivers and the working group, and it now requires further testing to determine its feasibility and acceptability and to identify additional areas for tool improvement. Developed with patient partners and knowledge users, the CPOT-Fam has the potential to (1) better support family caregivers who wish to contribute to the pain assessment process of their loved ones and (2) improve pain detection and timely pain management for critically ill patients in the ICU.
